# Unraveling the Complete Mitochondrial Genome of *Potamalpheops Sp.* (Purple Zebra Shrimp) (Crustacea: Decapoda) Provides Insights Into Its Phylogenetic Relationships and Gene Order Rearrangements

**DOI:** 10.1002/ece3.70546

**Published:** 2024-11-21

**Authors:** Sofia Priyadarsani Das, Yu‐Kai Kao, Huu‐The Nguyen, Yu‐Ru Lin, Zhen‐Hao Liao, Yeh‐Fang Hu, Fan‐Hua Nan

**Affiliations:** ^1^ Department of Aquaculture National Taiwan Ocean University Keelung Taiwan

**Keywords:** Alpheidae, gene‐rearrangement, mitogenome, phylogenomics, purifying selection

## Abstract

Understanding the functions of mitochondrial genomes is crucial for studies related to the evolution of genomes, phylogenomics, and species identification. For the first time, complete mitogenome of *Potamalpheops sp.* a non‐snapping shrimp has been successfully sequenced and characterized from Taiwan that belongs to the Decapoda order, Crustacea class, and Caridea infraorder. This study involved analysis of nucleotide composition, codon usage, gene ordering, evolutionary selection pressure, and comparative mitogenomics. The mitogenome of *Potamalpheops sp.* is 16,605 base pairs in length and consists of standard set of 37 genes found in metazoans. The gene rearrangements in the mitochondrial genome of this species shows extensive rearrangements comparing to the typical pattern found in pancrustaceans mitogenomes. Therefore, it could be concluded that gene rearrangements most likely happen only in the caridea infraorder. The current investigation discovered transposition of the tRNA and rRNA genes along with reversal in strands in the tRNAs. No other Alpheidae mitochondrial genome that has been investigated thus far has revealed this pattern. All 13 protein coding genes in the mitochondrial genomes of superfamily Alphoidea exhibited Ka/Ks values lower than 1, according to the ratios of nonsynonymous and synonymous substitutions rates. This suggests that a strong purifying selection had taken place. The maximum likelihood tree consisting of 46 mitogenomes of infraorder Caridea along with outgroups, revealed the existence of *Potamalpheops sp.* in the family Alpheidae and it formed a monophyletic group along with Palaemonoidea and Alpheoidea superfamily.

## Graphical abstract

*Potamalpheops sp.* is the species belong to family Alpheidae, and this is the first report from Taiwan. Also, the mitogenome of this species has been sequenced for the first time as there was a lack of sequence information regarding this species. The current species found to be in the family Alpheidae and support the pancrustacean mitogenome organization. The mitogenome consists of 16,605 bp and having novel gene re‐arrangements which is new to this family. There is also considerable purifying selection is found in the PCGs and it gives a slower rate of evolution to this family. Also, there are many structural variations is found in the control region. Moreover, the phylogenetic tree also corroborates with the findings and support the taxonomic position of this species in the family Alpheidae and is most close to Alpheus genus and found in the same clade. The current study can give an in‐depth knowledge about the species *Potamalpheops sp.* and also help in understanding its genetic structure also, there is need of having population genetic studies and nuclear genome analysis to get more information from this valuable species.

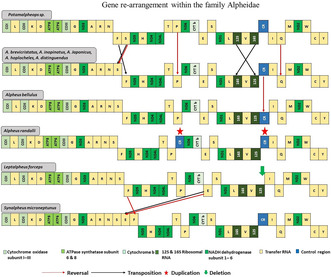

## 1 Introduction

Economically important order Decapoda, is a highly diversified groups within the crustaceans, consisting about 18,000 species, both living and extinct. This group includes many species such as crayfishes, lobsters, hermit crabs' shrimp, true crabs, and shrimps (Wang et al. 2020). Infraorder Caridea, under the order Decapoda is the group with the second highest level of diversity and having at least 13 super families and 34 families and many genera. Caridean shrimps (specifically the infraorder Caridea) display a wide range of variations in their physical characteristics, physiological processes, ecological interactions, and behavioral patterns (De Grave and Fransen 2011). There are currently about 2500 known species in 31 families within the Caridea. Among the caridean shrimps, the family Alpheidae is one of the most diversified and speciose family (Anker et al. 2006). Alpheid shrimps can be found in temperate, tropical, and subtropical marine environments worldwide. Their diversity rapidly reduces with depth, with only a few species exceeding 1000 m. It is concentrated in tropical shallow marine habitats, particularly on coral reefs, rubble flats, rocky coastlines, mangroves, seagrass beds, and mudflats. They are found in every type of aquatic environment on Earth, ranging from freshwater to anchialine cave‐dwelling pelagic marine species (Anker et al. 2005; Cai and Anker 2004).

The *Potamalpheops sp*. is the most underappreciated genus in the alpheid family. There are presently five species that are exclusively found in freshwater environments, despite the genus being primarily marine and brackish water despite the fact that the current species is thought to inhabit freshwater. There have been a few reports of this species' distribution from Asia. Powell (1979) established this genus from West Africa, where he found only three species: *P. haugi* (Coutière 1906), *P. pylorus* (Powell 1979), and *P. monodi* (Sollaud 1932). Because of this species' complexity and diversified distribution, additional research has not been done on it. Additionally, other species in this genus have been discovered in different regions of the globe, including Mexico ( *P. stygicola* ), Northern Australia ( *P. hanleyi* , *P. darwiniensis* ), New Caledonia ( *P. pininsulae* ), Southern Peninsular Malaysia and Singapore ( *P. amnicus* ), Western Indonesia and the Philippines ( *P. miyai* ), Singapore and northern Australia ( *P. tigger* ), Singapore ( *P. johnsoni* ), Sri Lanka ( *P. palawanensis* ), Vietnam (*P. kisi sp.n*.), and in this study, we found the species from Taiwan (*Potamalpheops sp*.) (Anker 2003; Bruce 1991; Bruce and Iliffe 1992; Christodoulou, Iliffe, and De Grave 2019; Hobbs Jr 1973; Yeo and Ng 1997).

Mitogenome has gained recognition being a crucial biomarker for studies on evolution due to its unique characteristics. It is inherited exclusively from the mother, has a remarkably compact genome, experiences an elevated mutation rate, and shows rapid rate of nucleotide substitution (Boore 1999; Taanman 1999; Wang et al. 2018). Decapoda order consist of circular mitogenome that ranges from 14 to 20 kb in size. They typically contain 13 protein‐coding genes (PCGs), two ribosomal RNA genes, 22 tRNA genes, and a non‐coding region known as the D‐loop or control region (Wang et al. 2020). Studying the mitogenome is crucial for understanding genome‐level characteristics and phylogenetic relationships among Decapoda order, as highlighted by several authors (Basso et al. 2017; Shen, Braband, and Scholtz 2013; Shen et al. 2012; Tan et al. 2018; Wang et al. 2019). In addition, the study of tRNAs and the unique gene arrangement in the mitogenome can provide valuable insights into phylogenetic relationships among these species (Tan et al. 2018). Furthermore, as mitochondria play a crucial role in generating 90% of cellular energy through the tricarboxylic acid cycle (TCA) and oxidative phosphorylation (OXPHOS), essential for any adaptation, they can offer valuable information about genetic advancements in metabolism for adaptability in Decapoda (Dhar et al. 2021; Sokolova 2018).

In this study, we obtained the comprehensive mitogenome of the non‐snapping shrimp *Potamalpheops sp*., as the complete mitogenome has not been reported yet. Our research primarily focusing on study of the nucleotide composition and codon usage patterns of genes that code for proteins, as well as investigation of selection pressure in PCGs. Furthermore, we analyze secondary structure of tRNA gene and also explore the possible structural changes in control region. In addition, we investigate the evolutionary relationship of *Potamalpheops sp*. with other caridean shrimps and some reference species using mitochondrial PCGs analysis.

## 2 Materials and Methods

### 2.1 Sample Collection, DNA Extraction, and Partial‐Genome Long‐Read Sequencing

The experimental organisms, only the wild caught were acquired from the southern region of Taiwan (*Potamalphleus sp*. purple zebra shrimp from Larma International Co. Ltd.). A total of 15 mature individuals were taken for the experiment, measuring between 8 and 15 mm in length. Following purchase, they were kept in a 27‐l acrylic aquarium (45 × 30 × 20 cm) with an air‐driven filter for filtration and oxygen delivery. The salinity was kept at 15‰, the water temperature was kept at 26°C, and a light regime of L12:D12 (L for light, D for dark) was offered. We gave them one daily meal of Hai Feng Crystal red shrimp feed, making sure to remove any leftovers and excrement before each feeding. We changed the water every 3 days, replacing one‐third of the total volume each time. The main method used to identify the specimen was to look at its morphological features. The university's ethical norms for the use and care of animals were closely followed when handling the shrimp. Fifteen individuals were put to death by cold shock, and the whole animal was preserved in 95% ethanol at −80°C for further DNA isolation and sequencing.

The total DNA was extracted from the whole body of 15 individuals using the Qiagen DNeasy Blood & Tissue Kit (QIAGEN Inc. Germantown, MD) according to the manufacturer's instruction. The extracted DNA's quality (260/280 ratio) has been analyzed using NanoDrop one (Thermo Scientific). Also, the DNA quality and intactness was evaluated via 0.8% agarose gel electrophoresis.

Approximately 10 μg of DNA from a single individual has been outsourced to National Taiwan Ocean University (Prof. Ying‐Ning Ho, Institute of Marine Biology) for sequencing in Oxford Nanopore Technology (ONT)‐MinION platform. Whole‐genome long‐read libraries were obtained using a sequencing kit by Oxford Nanopore Technologies Ltd. (ONT, SQK‐LSK114) with manufacturer's instruction. The library was loaded in MinION FLO‐MIN114 flowcells, controlled by MinKNOW v22.07.9. The raw data was converted to FASTQ format using Dorado v0.3.4 in super accuracy mode (SUP), generating ~2 GB high data (> Q10).

### 2.2 Mitochondrial Genome Assembly of *Potamalpheops Sp.* and Annotation

A total of 899,305 ONT reads generated from Nanopore sequencing using MinION. The reads were *denovo* assembled in the CLC Genomics Workbench Version 23.0.4 (https://www.qiagenbioinformatics.com/products/clc‐genomics‐workbench/). The reads, more than 16 kb in size were blasted in NCBI‐nr database and most of the contigs were match with complete mitogenome of *Alpheus japonicus*. Then the reference‐based mapping of the contigs were done by taking complete mitogenome of *Alpheus japonicus* with accession number NC_038116.1 submitted from China as a reference sequence. Then, based on the similarity index the complete mitogenome of *Potamalpheops sp*. (16,605 bp) has been curated and sequence preprocessing has been done manually in Bioedit v.7.2.5 and the processed sequence was annotated using MITOS online web server in Galaxy portal (https://mitos.bioinfuni‐leipzig.de/), the ExPASy translate tool (https://web.expasy.org) (Gasteiger et al. 2003) was used to predict the ORFs (open reading frame) to confirm the missing stop codons. The overlapping regions and intergenic spacers between the two genes were analyzed using Bioedit v.7.2.5 (Hall 1999) and Microsoft Excel manually. The annotated mitogenome has submitted in the GenBank with accession number OR825035. The circular genome has been visualized using the OG Draw webserver (Greiner, Lehwark, and Bock 2019), and the tRNAScan‐SE software (Lowe and Eddy 1997) has been used to confirm the identity of tRNA genes and the anticodons using the invertebrate mitochondrial genetic code method. Also, MiTFi software (Jühling et al. 2011) was used to confirm the identified and predicted secondary structure of tRNA genes embedded in the MITOS web server. Visualization of tRNA cloverleaf structure was done using Forna webserver (http://rna.tbi.univie.ac.at/forna/) (Kerpedjiev, Hammer, and Hofacker 2015). MEGA X (Kumar et al. 2018) was used to detect the nucleotide composition and codon usage of the PCGs. The base skewness was determined by AT skew = [A−T]/[A+T] and GC skew = [G−C]/[G+C] (Perna and Kocher 1995). Possible microsatellite (Simple Sequence Repeats, SSRs) and different types of tandem repeats were identified in the control region by Microsatellite repeat finder (http://insilico.ehu.es/mini_tools/microsatellites/?info) web tools and the web server Tandem Repeat Finder v. 4.09 (http://tandem.bu.edu/trf/trf.html) (Benson 1999). A comprehensive analysis was conducted on the secondary structure along with the stem loops and hairpin loop in the control region of *Potamalpheops sp*. using RNA structure web server and mfold webserver (http://rna.urmc.rochester.edu/RNAstructureWeb/Servers/Predict1/Predict1.html; http://www.unafold.org/results/6/24Jul29‐06‐48‐31/) with default parameters and with temperature of 37°C was used for the prediction.

### 2.3 Phylogenetic Analysis

To investigate the phylogenetic relationship among the infraorder Caridea which consists of fresh/marine water snapping and non‐snapping shrimps were used for reconstructing the phylogenetic relationship among these species. The mitogenome data of 45 individuals belonging to eight super families (Alpheoidea, Palaemonoidea, Pandaloidea, Atyoidea, Oplophoroidea, Nematocarcinoidea, Penaeoidea, and Bresilioidea) and outgroups (Brachyura and Stenopodidea) were downloaded from NCBI (www.ncbi.nlm.nih.gov) (Table 3). The nucleotide sequences of 13 PCGs were manually edited, concatenated, and converted into amino acid sequences for generation of maximum likelihood algorithm (ML) tree in MEGA X (Kumar et al. 2018). The bootstrap value that employed for the ML‐based tree was 1000 replicates. The nucleotides adenine, guanine, cytosine, and thymine were used, and generalized GTR substitution rates were taken into account.

### 2.4 Comparative Mitogenomics and Gene Arrangement Analysis in Superfamily Alphoidea

Comparison of mitochondrial gene synteny and homology among the most diverse superfamily Alphoidea of infraorder Caridea was done to investigate possible rearrangements within the family using the CREx software (http://pacosy.informatik.uni‐leipzig.de/crex) (Bernt et al. 2007). The gene rearrangement events encompass transposition, reversal, reverse transpositions, and tandem‐duplication‐random‐loss. Also, the nucleotide composition and codon usage of the *Potamalpheops sp*. (Table 4 and Figure 2) was compared to investigate the fundamental and distinct characteristics of their mitogenomes. The conserved variable site ration, K2P distance, and Ka/Ks substitution ration among 13 PCGs were calculated using MEGA X (Kumar et al. 2018).

## 3 Results

### 3.1 Mitogenome and Its Organization

**FIGURE 1:**
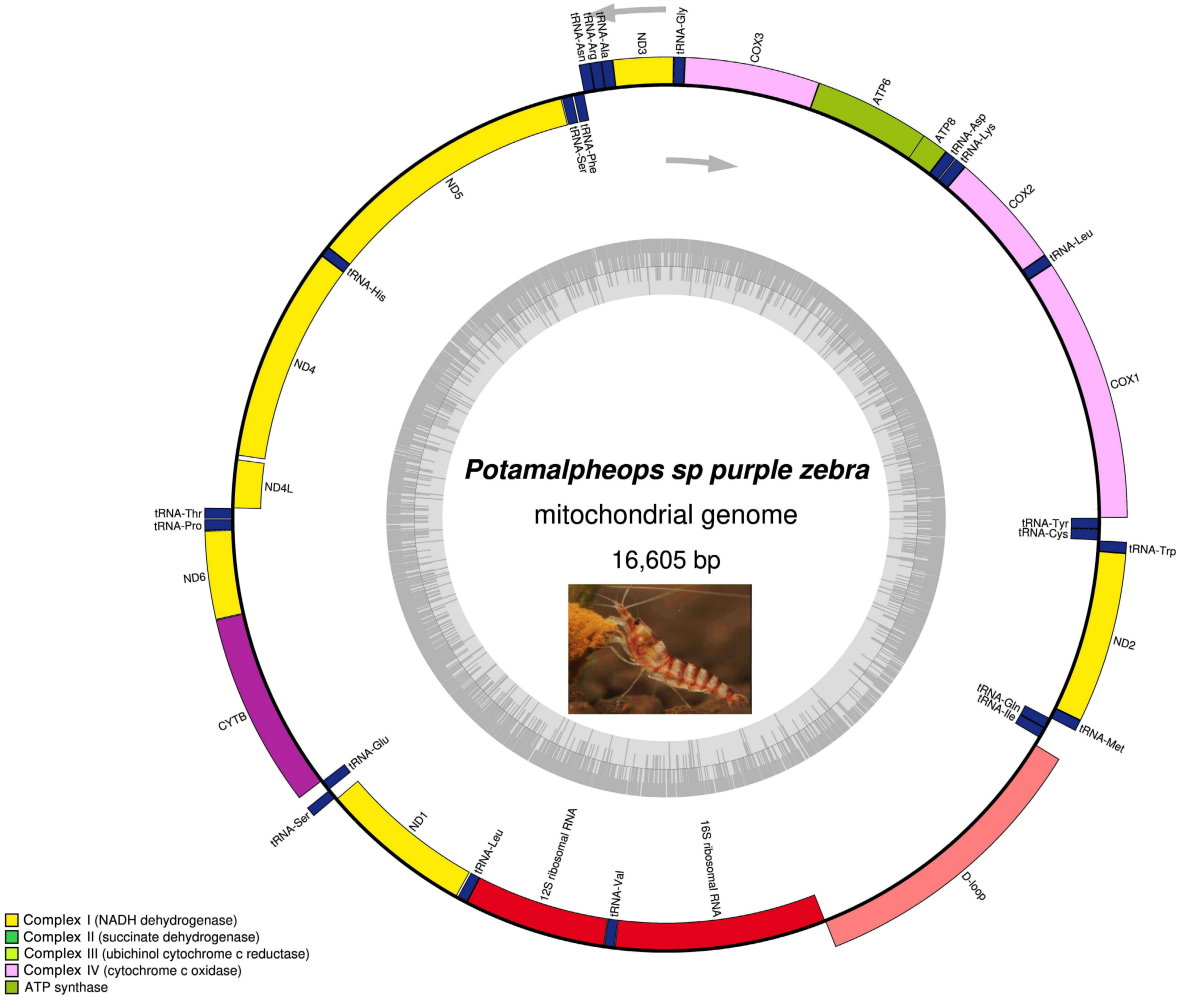
The mitogenome of the *Potamalpheops sp*. is represented by a circular map. The genes located on the inner circle are transcribed in a clockwise direction, while the genes on the outer circle are transcribed in a counterclockwise fashion. Distinct genetic variants are represented by solid boxes of varying hues. The genes ND1‐6 are highlighted in the color yellow, whereas COX 1–3 are highlighted in a light shade of purple. ATP6 and 8 are marked in green, CYTB in purple, tRNA genes in blue, rRNAs in red, and the control region in peach.

The mitogenome of *Potamalpheops sp*. consisted of a double‐stranded circular DNA fragment of 16,605 bp in length (Genbank accession: OR825035). Like other shrimp species, it consists of 37 genes (22tRNA genes, 13 PCGs, and 2 rRNA genes) and an AT rich control region (Figure 1 and Table 1) (Ennis et al. 2021). In the current mitogenome there were 15 intergenic spacer stretches of 1–48 bp between neighboring genes and three overlaps of 1–5 bp (Table 1). There is a 48 bp long spacer found between D‐loop and tRNA‐Ile and a 24 bp long spacer found between tRNA‐His to ND4 gene and the overlap of 5 bp is found in between tRNA‐Asp to ATPase‐8 gene.

**TABLE 1:** Genomic features of the complete mitogenome of *Potamalpheops sp*.

Locus name	Start	Stop	Strand	Size (bp)	Anticodon	Start/stop	Intergenic nucleotides
COI	1	1536	+	1536	—	TCG/TAA	0
tRNA‐Leu	1537	1603	+	67	TAG	—	0
COII	1604	2287	+	684	—	ATG/TAA	0
tRNA‐Lys	2288	2357	+	70	TTT	—	3
tRNA‐Asp	2361	2426	+	66	GTC	—	0
ATP8	2427	2585	+	159	—	ATA/TAA	−5
ATP6	2579	3256	+	678	—	ATG/TAA	−1
COIII	3256	4042	−	787	—	ATG/T..	0
tRNA‐Gly	4043	4106	+	64	TCC	—	0
ND3	4107	4458	+	352	—	ATA/T..	0
tRNA‐Ala	4459	4523	+	65	TGC	—	0
tRNA‐Arg	4524	4588	+	65	TCG	—	0
tRNA‐Asn	4589	4654	−	66	GTT	—	0
tRNA‐Phe	4655	4719	−	65	GAA	—	8
tRNA‐Ser	4728	4793	−	66	TCT	—	4
ND5	4798	6516	−	1719	—	CTA/…	0
tRNA‐His	6517	6580	−	64	GTG	—	0
ND4	6581	7919	−	1339	—	AAT/T..	24
ND4L	7944	8243	−	300	—	TTA/T..	0
tRNA‐Thr	8244	8306	+	63	TGT	—	4
tRNA‐Pro	8311	8375	+	65	TGG	—	1
ND6	8377	8891	+	515	—	ATA/TA.	1
CYTB	8893	10,028	+	1136	—	ATG/TA.	2
tRNA‐Glu	10,031	10,097	−	67	TTC	—	2
tRNA‐Ser	10,100	10,160	−	61	TGA	—	9
ND1	10,170	11,117	−	948	—	TTA/T..	9
tRNA‐Leu	11,127	11,191	−	65	TAA	—	0
12S rRNA	11,192	12,073	−	882	—	—	1
tRNA‐Val	12,075	12,139	−	65	TAC	—	1
16S rRNA	12,141	13,447	−	1307	—	—	0
D‐loop	13,448	15,150	+	1703	—	—	48
tRNA‐Ile	15,199	15,265	−	67	GAT	—	0
tRNA‐Gln	15,266	15,333	−	68	TTG	—	3
tRNA‐Met	15,337	15,402	+	66	CAT	—	0
ND2	15,403	16,401	+	999	—	ATT/TAA	−1
tRNA‐Trp	16,401	16,467	+	67	TCA	—	0
tRNA‐Cys	16,468	16,537	−	70	GCA	—	0
tRNA‐Tyr	16,538	16,605	−	68	GTA	—	—

*Note:* The table contains start and end positions of each gene, intergenic nucleotide size, gene size, start and stop codons, anticodons for tRNAs, and strand information. For intergenic nucleotide, positive and negative values indicate overlap and gaps between adjacent genes, respectively. In strand column, + and − represents the positive and negative strands, respectively.

**FIGURE 2:**
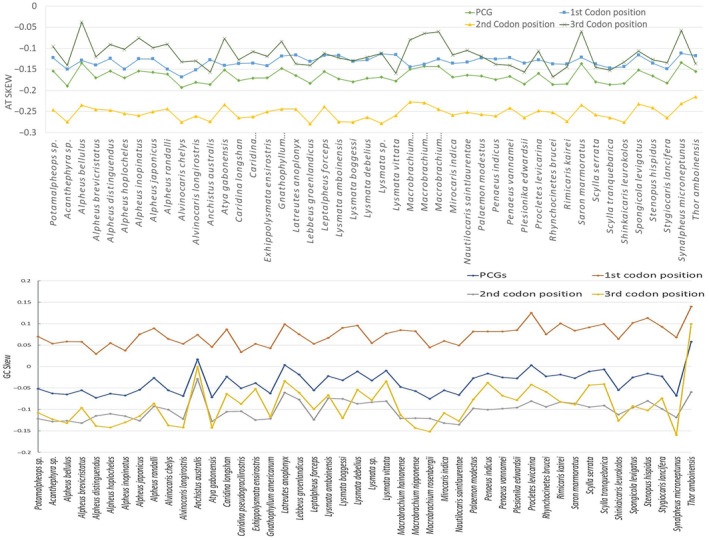
Comparative analysis of AT and GC Skew of *Potamalpheops sp*. with individuals from Infraorder Caridea and other reference species.

**FIGURE 3:**
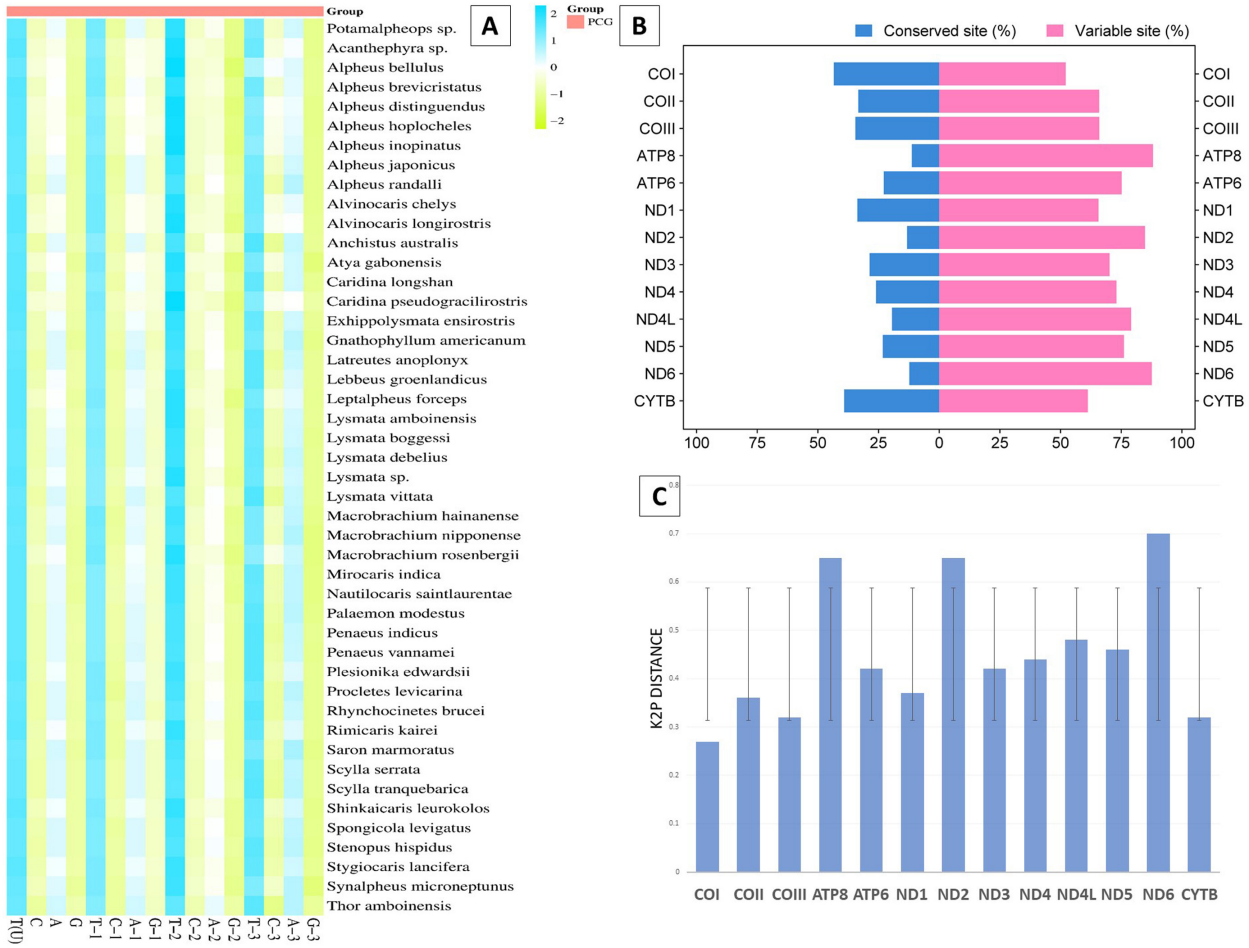
(A) Heatmap of nucleotide composition at different codon positions of concatenated PCGs. Color scale indicates A, T, G, and C content at different codon positions of PCGs of each species (B) Conserved and Variable regions (C) K2P distances among the 13PCGs.

A substantial A+T bias was observed in the mitogenome of *Potamalpheops sp*. (65.1%). The highest level of significance was observed in the control region with a value of 77.9%. This was followed by rRNAs at 68.6%, tRNAs at 65.8%, and the lowest level of significance was found in PCGs at 62.4% (Table 2). In this study, the AT‐skew showing a range from −0.15 to 0.03, whereas GC‐skew shows range from −0.27 to 0.32. The GC‐skew values were mostly negative and positive GC‐skew can be seen in rRNA and tRNA regions (Table 2). The overall AT and GC Skew of the 46 different shrimp and crab species is shown in Figure 2. Also, the nucleotide composition along with all the codon positions (1st, 2nd, and 3rd codon positions) being analyzed and it shows every species mitogenome PCGs are AT rich (Figure 3A). The percentages of conserved and variable sites revealed that ATPase 8, ND2 and ND6 were the most variable among the PCGs (> 75%) and COI and Cyt b found to be the most conserved PCGs (> 30%) which is also shows the same result with K2P genetic distance where ATPase 8, ND2, and ND6 genes shows most diverged among 13 PCGs (Figure 3B,C).

**TABLE 2:** Nucleotide base composition and skewness of different regions of the mitogenome of *Potamalpheops sp*.

*Potamalpheops sp*. Mitogenome	Size (bp)	A%	T%	G%	C%	A+T%	AT‐skew	GC‐Skew
Whole	16,605	35.7	29.4	12.6	22.3	65.1	0.09	−0.27
PCGs	11,152	26.4	36	17.9	19.8	62.4	−0.15	−0.05
tRNAs	1450	33.9	31.9	18.7	15.5	65.8	0.03	0.09
rRNAs	2189	30.2	38.4	20.8	10.6	68.6	−0.11	0.32
Control region	1703	40.8	37.1	9.5	12.7	77.9	0.04	−0.14

### 3.2 PCGS and Codon Usage

**FIGURE 4:**
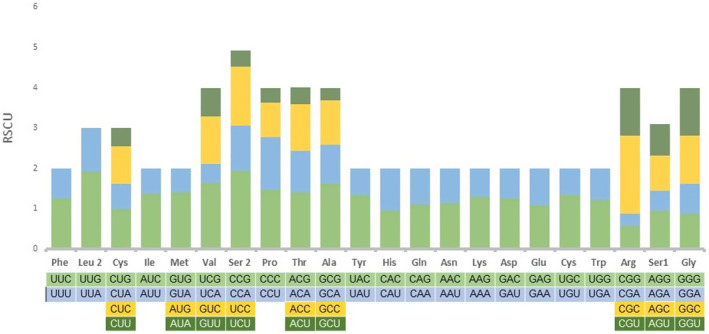
Relative synonymous codon usage (RSCU) of the PCGs in the *Potamalpheops sp*. mitogenome. Different color indicates the corresponding codons.

The cumulative length of the 13 PCGs in the mitogenome of *Potamalpheops sp*. was 11,152 base pairs (bp), representing 67.16% of the whole sequence. The majority of PCGs in *Potamalpheops sp*. initiated by the normal start codon ATN, with four genes using ATG, three using ATA, and one using ATT. However, COI had an unusual putative codon TCG as its start codon (Table 1). Regarding stop codons, COIII, ND3, ND4, ND4L, ND1 utilized a solitary T residue to halt transcription, while five genes (COI, COII, ATP8, ATP6, and ND2) concluded with the standard stop codon TAA, two genes (ND6 and CYTB) having the stop codon TA and lastly an incomplete stop codon is found in ND5 gene (Table 1). The analysis of the relative synonymous codon usage (RSCU) in *Potamalpheops sp*. revealed that the six most frequently utilized codons were UUU (Phe), AUU (Ile), UUA (Leu1), UUC (Phe), AUA (Ile), and UAU (Tyr), with counts of 200, 179, 162, 119, and 107, respectively. Conversely, the least commonly used codons were CGU (Arg), CGC (Arg) with a count of 9 and 5, respectively (Figure 4 and Table 3).

**TABLE 3:** The codon counts and relative synonymous codon usages in 13 PCGs of *Potamalpheops sp.* mitogenome.

Amino acids	Codon	Count	RSCU	Amino acids	Codon	Count	RSCU	Amino acids	Codon	Count	RSCU	Amino acids	Codon	Count	RSCU
Phe	UUU(F)	200	1.25	Ser 2	UCU(S)	90	1.94	Tyr	UAU(Y)	107	1.32	Cys	UGU(C)	46	1.33
	UUC(F)	119	0.75		UCC(S)	52	1.12		UAC(Y)	55	0.68		UGC(C)	23	0.67
Leu 2	UUA(L)	162	1.91		UCA(S)	68	1.47	Stop	UAA(*)	69	1.05	Trp	UGA(W)	58	1.21
	UUG(L)	92	1.09		UCG(S)	18	0.39		UAG(*)	62	0.95		UGG(W)	38	0.79
Leu 1	CUU(L)	83	0.98	Pro	CCU(P)	63	1.47	His	CAU(H)	40	0.94	Arg	CGU(R)	9	0.56
	CUC(L)	53	0.63		CCC(P)	56	1.31		CAC(H)	45	1.06		CGC(R)	5	0.31
	CUA(L)	80	0.94		CCA(P)	36	0.84	Gln	CAA(Q)	48	1.1		CGA(R)	31	1.94
	CUG(L)	38	0.45		CCG(P)	16	0.37		CAG(Q)	39	0.9		CGG(R)	19	1.19
Ile	AUU(I)	179.1	1.36	Thr	ACU(T)	66	1.41	Asn	AAU(N)	77	1.14	Ser1	AGU(S)	44	0.95
	AUC(I)	85	0.64		ACC(T)	48	1.03		AAC(N)	58	0.86		AGC(S)	23	0.5
Met	AUA(I)	112	1.4		ACA(T)	54	1.16	Lys	AAA(K)	60	1.29		AGA(R)	40	0.86
	AUG(M)	48	0.6		ACG(T)	19	0.41		AAG(K)	33	0.71		AGG(R)	36	0.78
Val	GUU(V)	76	1.63	Ala	GCU(A)	71	1.62	Asp	GAU(D)	54	1.26	Gly	GGU(G)	49	0.88
	GUC(V)	22	0.47		GCC(A)	42	0.96		GAC(D)	32	0.74		GGC(G)	41	0.73
	GUA(V)	55	1.18		GCA(A)	48	1.1	Glu	GAA(E)	49	1.09		GGA(G)	68	1.21
	GUG(V)	33	0.71		GCG(A)	14	0.32		GAG(E)	41	0.91		GGG(G)	66	1.18

### 3.3 tRNA Genes, rRNA Genes, and the Control Region

**FIGURE 5:**
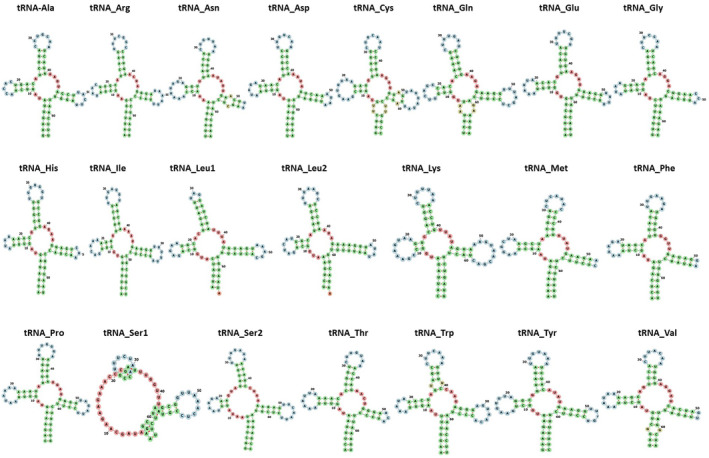
Cloverleaf secondary structure of 22 tRNAs of *Potamalpheops sp*. visualized in the Forna web server.

The cumulative length of the 22 transfer RNA genes in *Potamalpheops sp*. was 1450 base pairs, representing 8.73% of the whole mitogenome. The total length of tRNAs were corroborate with the majority of the caridean mitochondrial genomes analyzed have a length of 1445–1451 base pairs. The lengths of the different sequences varied, with the smallest being tRNA‐Thr at 63 base pairs and the largest being tRNA‐Lys and tRNA‐Cys at 70 base pairs (Table 1). The anticipated secondary structures were displayed in Figure 5. All tRNAs can adopt the characteristic cloverleaf secondary structure, with the exception of tRNAS1 which lacks the usual DHU arm.

The combined length of the two ribosomal RNA genes, including 16S rRNA (1307 bp) and 12S rRNA (882 bp) covers 2189 bp. This accounts for 13.18% of the complete mitogenome, as seen in Table 2. The 12S rRNA gene was positioned between trnL1 and trnV, whereas the 16S rRNA gene was situated between trnV and the control region. In both cases, there were one each intergenic spacers or overlaps found 12SrRNA > tRNA‐Val > 16SrRNA. Both rRNA genes are located on the antisense strand (Figure 1).

**FIGURE 6:**
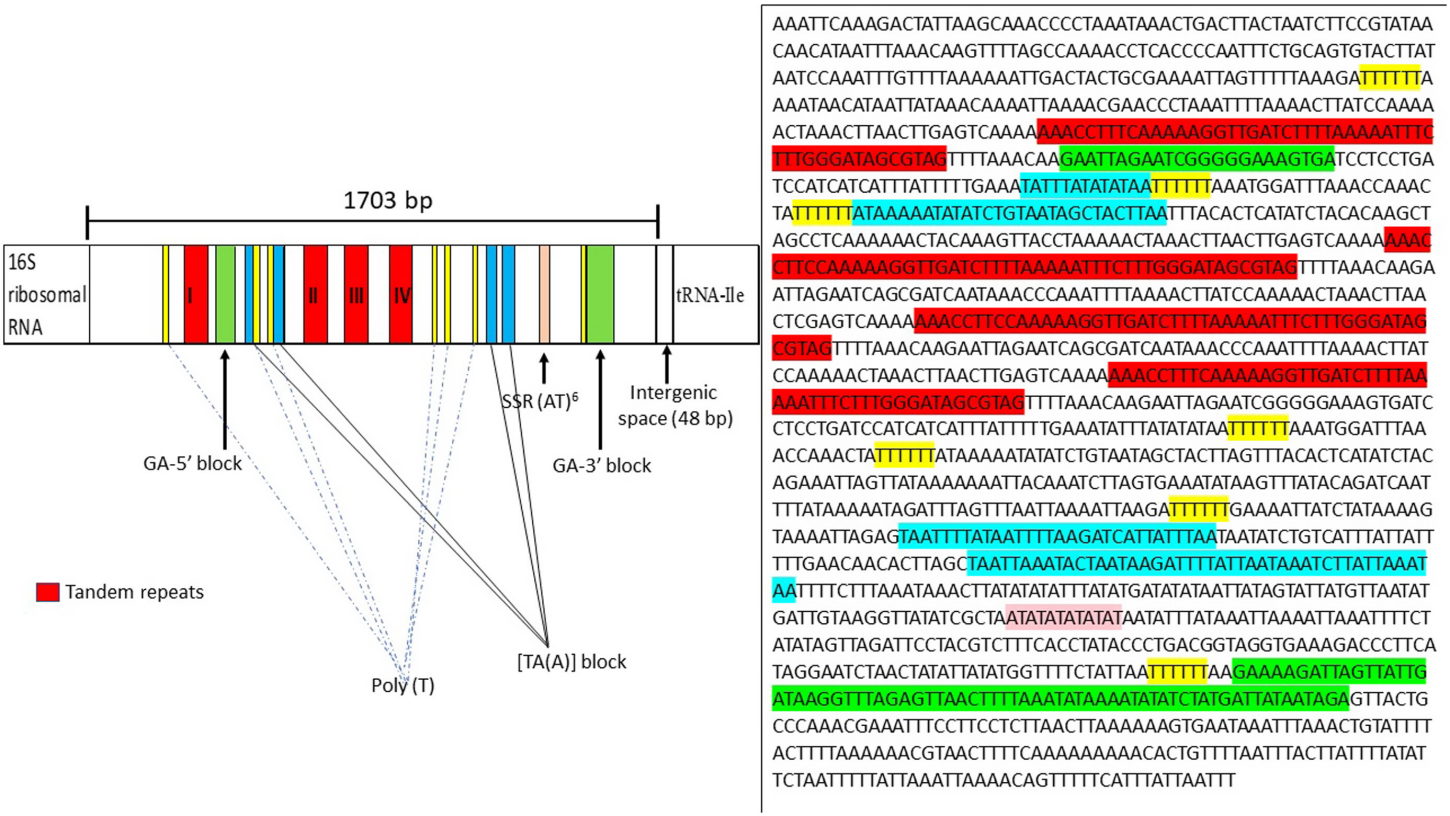
Structural organization of the mitochondrial control region of *Potamalpheops sp*. along with the sequence each color represent its adjacent blocks. The putative conserved regions are highlighted: I, II, III, and IV represents the tandem repeats found four times in the control region shown in red color. Two GA blocks (GA‐5′ block and GA‐3′ block) are marked in green. The poly T stretch is marked in yellow. The [TA(A)] n‐blocks are marked with sky blue color. Other elements are highlighted as shown in the figure.

The control region of *Potamalpheops sp*. had a length of 1703 base pairs and was situated between the 16S rRNA and tRNA‐Ile genes (Table 1). The tandem repeat finder analysis revealed a fragment of 35 bp in length and repeated four times between positions 1–791 bp. The 1703 bp control region also having a (AT)_9_ microsatellite. The fragment was also having GA‐5′ and GA‐3′ blocks along with four [TA(A)] n‐blocks and 5 poly T stretches (Figure 6). Also, a 48 bp overlap was found between control region and tRNA‐Ile.

**FIGURE 7:**
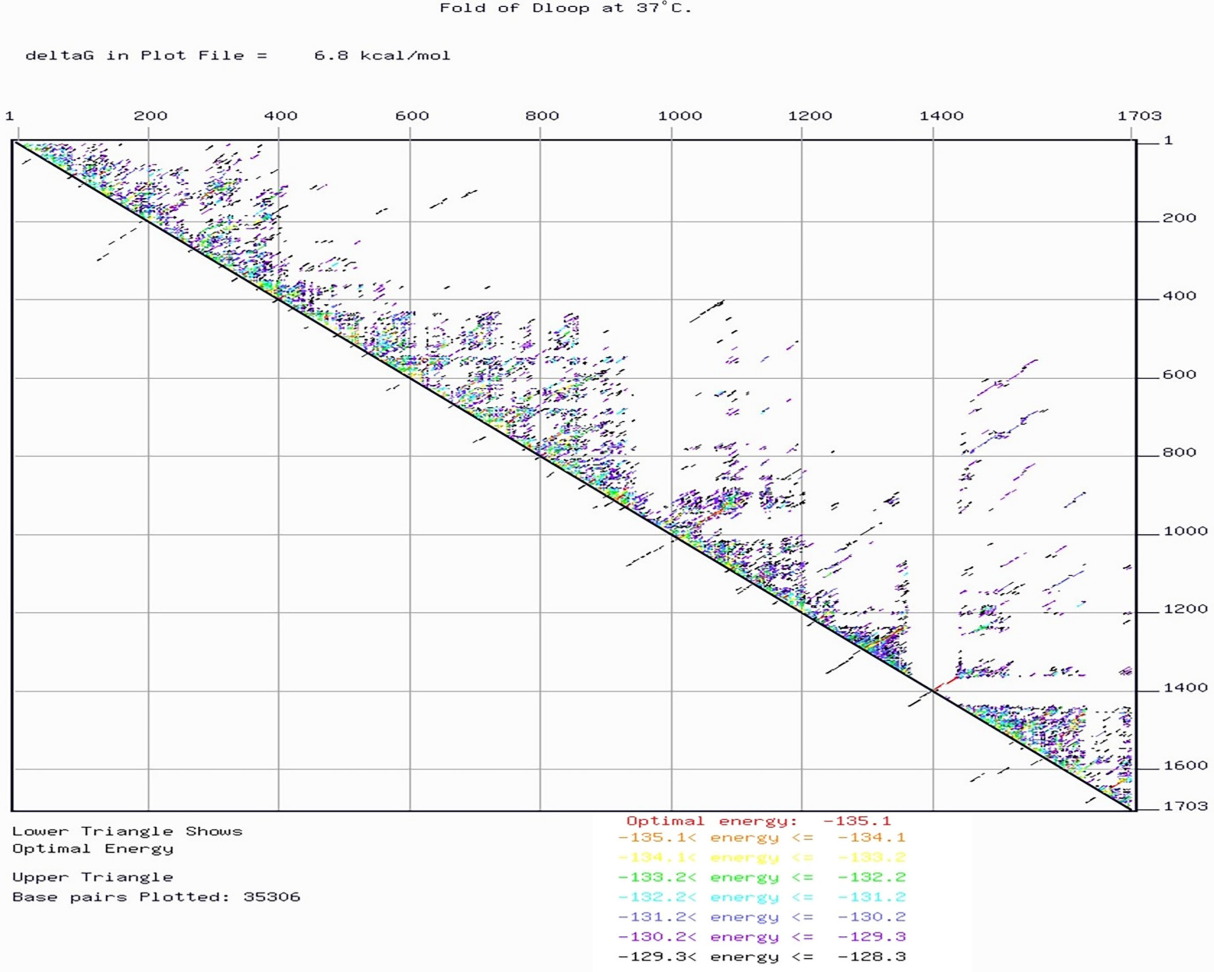
Dot plot showing the D‐loop hairpin loop formation at 37°C.

The mfold webserver was used to predict the secondary structure which generated a configuration with a minimum achievable free energy (change in Gibbs free energy [ΔG] = −129.05 kcal/mol to −135.14 kcal/mol). This prediction was obtained considering a default melting temperature of 37°C with other default settings. This exhibited several stem‐loop structures distributed across the region with dot plots (Figure 7).

### 3.4 Selection Pressure

**FIGURE 8:**
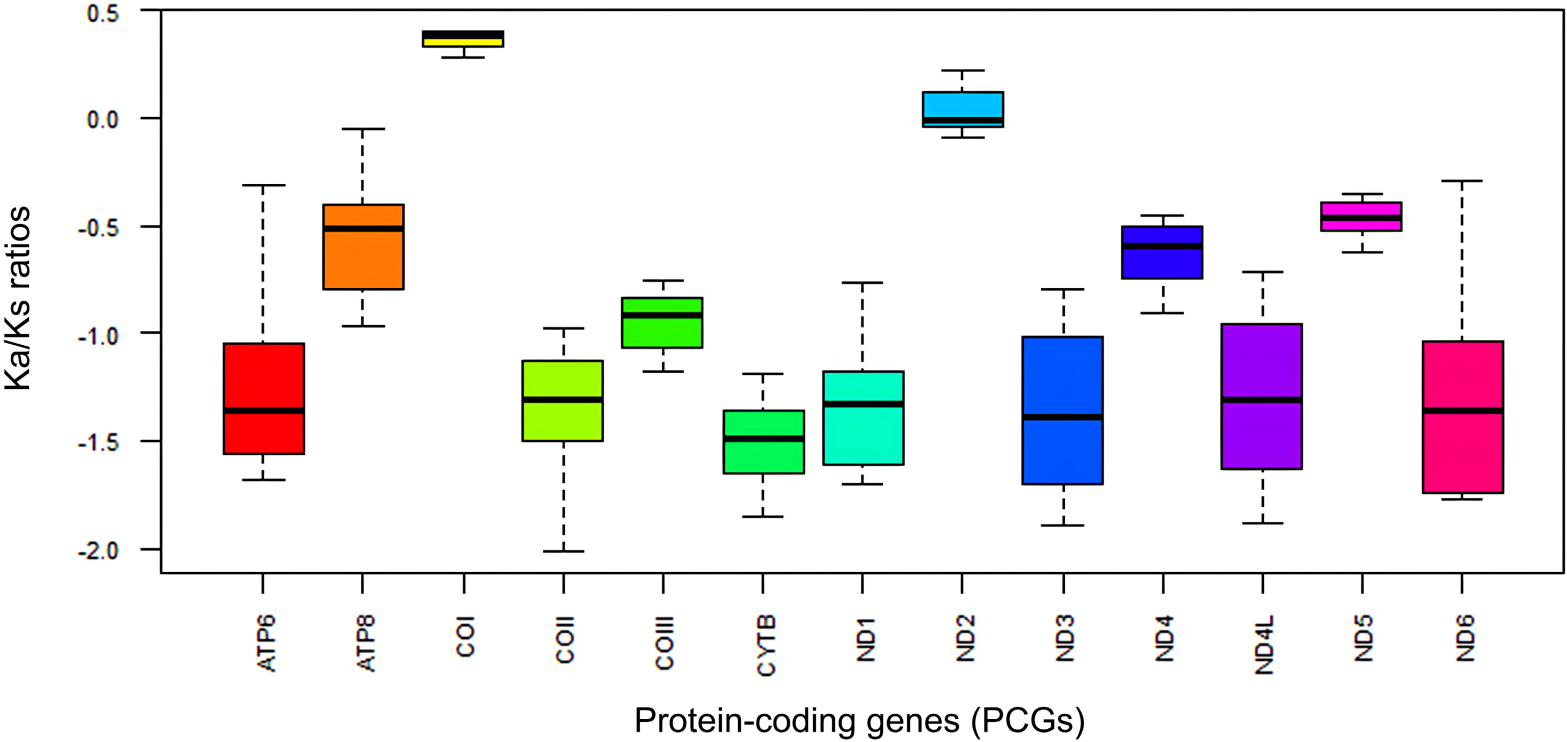
The Ka/Ks values of the 13 mitochondrial PCGs in 19 individuals of superfamily Alphoidea.

The selection pressure analysis has been done by taking into account the Ka/Ks ration which estimates the evolution of non‐synonymous substitution to synonymous substitution particularly in PCGs. In the current study, the Ka/Ks values for 13 PCGs were < 1 (−1.549 to 0.346) indicating the presence of purifying selection in the superfamily Alphoidea. The lowest Ka/Ks ration is observed in Cytochrome b (Cytb) (−1.549) indicating the gene had slowest rate of evolution and the highest observed in COI (0.346) showing a fastest rate of evolution (Figure 8). Only two genes namely COI and ND2 shows the result more than 0 but other than that each of the genes Ka/Ks ratios are < 0.

### 3.5 Gene Rearrangements

**FIGURE 9:**
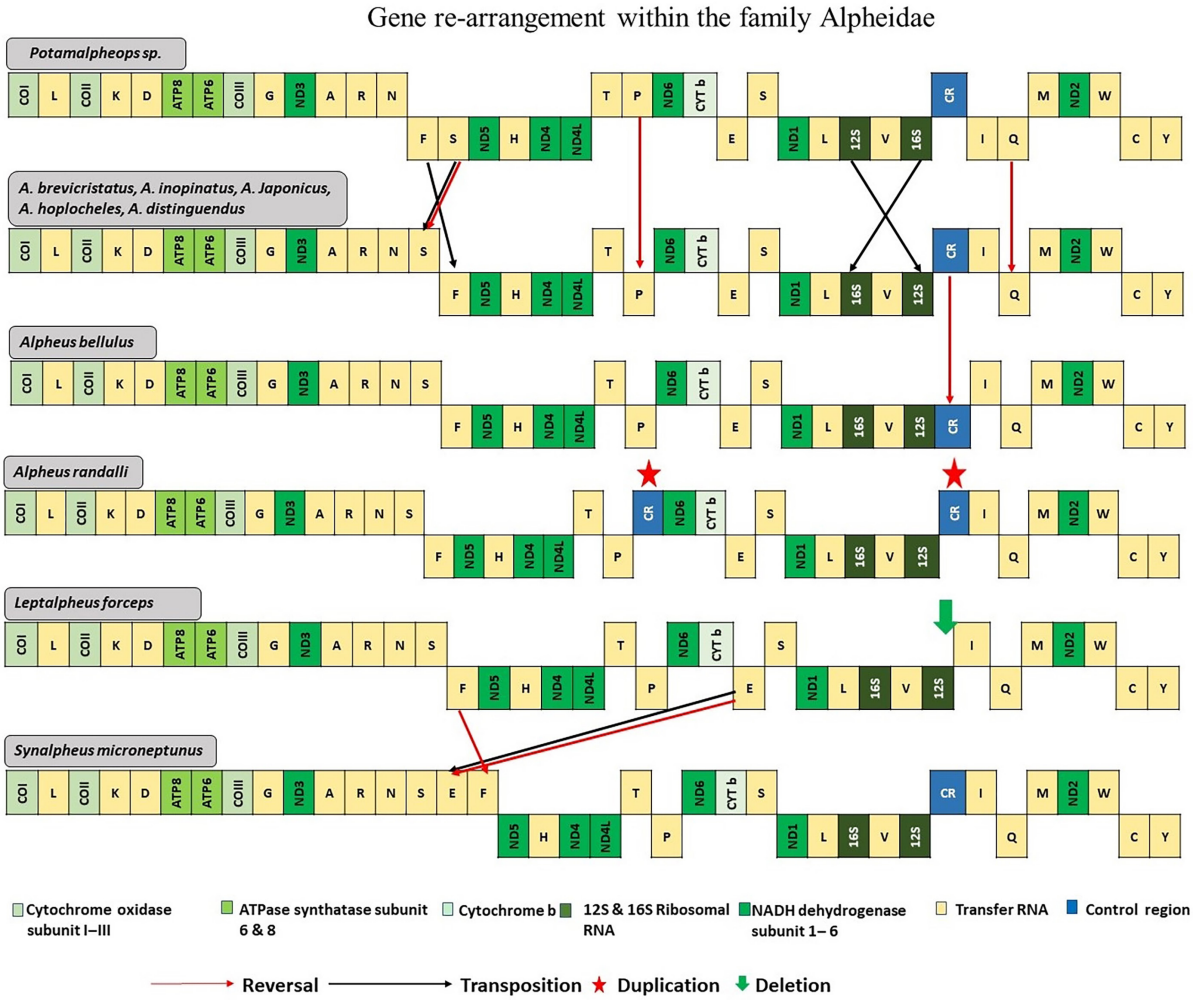
Mitogenome organization and gene re‐arrangement illustrating gene transposition, reversal, deletion and duplication within the family Alpheidae. Each color represents the different genes: Light to dark shades of green represents the PCGs and rRNA genes. Blue color represents control region and light yellow color represents the tRNAs. The reversal represented by the red arrow, Transposition represented by black arrow, deletion represented by a down word green arrow and red star mark represents the duplicated genes.

The gene arrangement pattern is unique and slightly differ from other alpheid shrimps, the mitogenome of *Potamalpheops sp*. encoded 8 PCGs and 12 tRNAs in positive strand and other 5 PCGs, 10 tRNAs and 2 rRNAs are in negative strand (Figure 9) Whereas, in most of the alpheids 9 PCGs and 13 tRNAs encoded in positive strand and 4 PCGs, 2 rRNAs, and 9 tRNAs in negative strands (Chak, Baeza, and Barden 2020; Ennis et al. 2021; Scioli, Plouviez, and Felder 2020; Shen et al. 2012; Wang et al. 2020; Zhong, Zhao, and Zhang 2019). In our study, we discovered that the mitochondrial genome of *Potamalpheops sp*. exhibited two instances of transposition involving tRNA‐Phe, tRNA‐Ser1, 12S rRNA, and 16S rRNA, leading to a distinct arrangement of rRNA genes in comparison to other mitogenomes. Additionally, we observed reversal of two tRNAs (Ser, Ile) from positive to the negative strand and tRNA‐Phe from negative strand to positive strand (Figure 9).

### 3.6 Mitogenome Phylogeny

**FIGURE 10:**
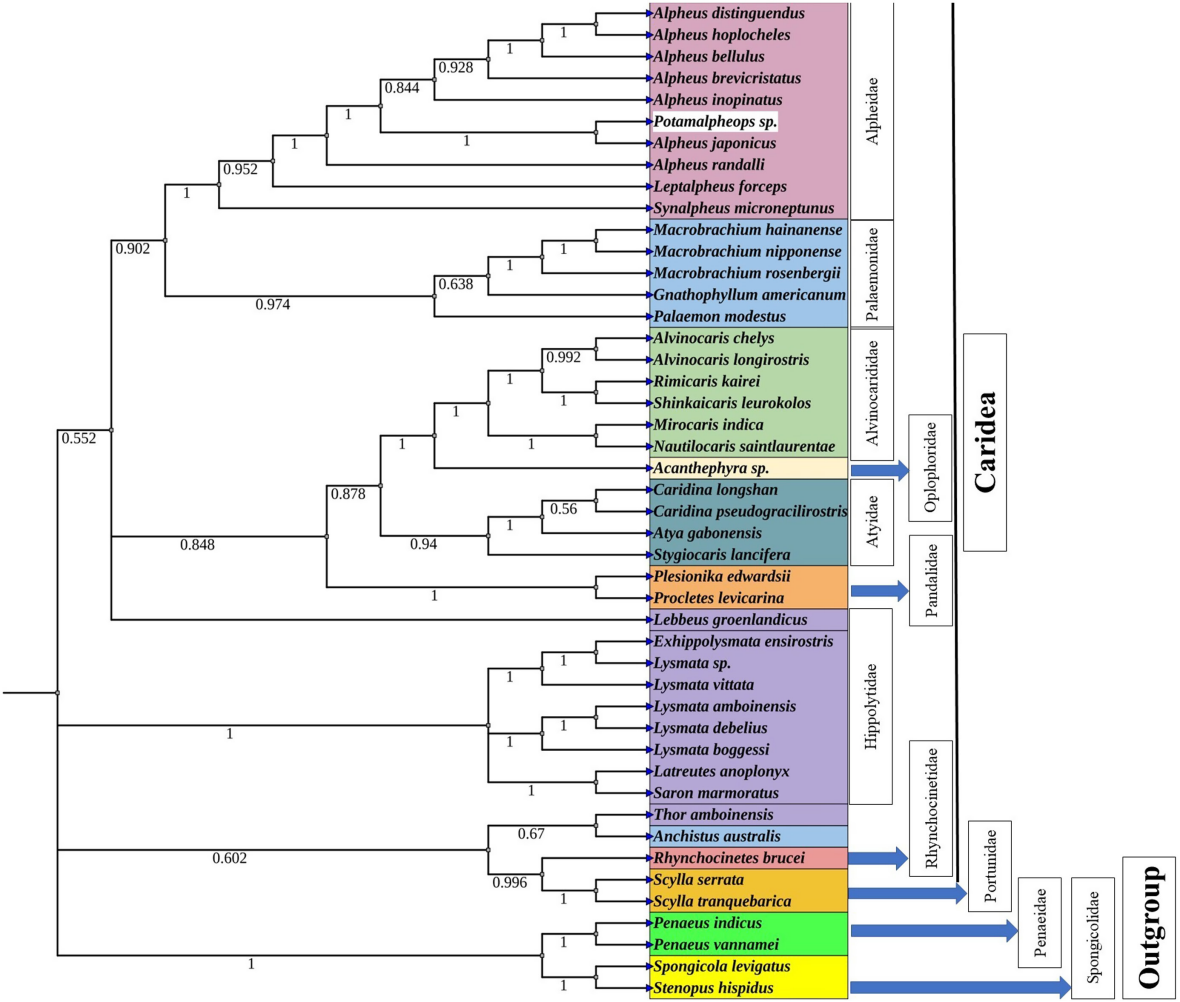
Maximum‐likelihood phylogenetic tree based on the amino acid alignments of 13 protein coding genes in the mitochondrial genome of *Potamalpheops sp*. and 45 other decapoda. Outgroups included two species of crabs (*Scylla serrata* and *S. tranquebarica*), two species of stenopodid shrimps (*Stenopus hispidus* and *Spongicola levigatus*) and two species of prawns (*Penaeus vannamei* and *P. monodon*). Number at each node represents bootstrap values.

This study aimed to further understand the evolutionary relationships within the infraorder Caridea. In the present analysis, collection and analysis of the combined nucleotide sequences of 13 PCGs from 42 different Caridea species with two species each from infraorder Brachyura and Stenopodidea as outgroup (Table 4). The caridea infraorder consists of eight superfamily (Alpheoidea, Atyoidea, Bresilioidea, Nematocarcinoidea, Oplophoroidea, Palaemonoidea, Pandaloidea, and Penaeoidea). The phylogenetic trees obtained from maximum likelihood (ML) analyses exhibited some diverse topologies, as shown in Figure 10. As the tree was built after concatenation of PCGs and rearrangement of the genes the distance and similarity of the genomes based one the nucleotide diversity only.

**TABLE 4:** Summary of taxonomic position of mitogenomes of different Decapoda along with their mitogenome length and NCBI accession number.

Sl No.	Infra Order	Superfamily	Family	Taxa	Length	Accession number
1.	Caridea	Alpheoidea	Alpheidae	*Potamalpheops sp*.	16,605	OR825035
2.				*Alpheus brevicristatus*	15,705	NC_079948
3.				*Alpheus inopinatus*	15,789	NC_041151
4.				*Alpheus japonicus*	16,619	NC_038116
5.				*Alpheus hoplocheles*	15,735	NC_038068
6.				*Alpheus distinguendus*	15,700	NC_014883
7.				*Alpheus randalli*	15,676	MH796168
8.				*Alpheus bellulus*	15,738	MH796167
9.				*Leptalpheus forceps*	15,463	MN732884
10.				*Synalpheus microneptunus*	15,603	NC_047307
11.			Hippolytidae	*Latreutes anoplonyx*	16,420	NC_081991
12.				*Lysmata sp*.	16,758	MW836830
13.				*Lysmata boggessi*	17,345	NC_064049
14.				*Lysmata amboinensis*	16,735	NC_050676
15.				*Lysmata vittata*	22,003	NC_049878
16.				*Lysmata debelius*	16,757	NC_060421
17.				*Lebbeus groenlandicus*	17,399	NC_045223
18.				*Exhippolysmata ensirostris*	16,350	MK681888
19.				*Saron marmoratus*	16,330	NC_050677
20.				*Thor amboinensis*	15,553	NC_051930
21.		Atyoidea	Atyidae	*Atya gabonensis*	15,978	OP650929
22.				*Caridina pseudogracilirostris*	15,451	NC_079936
23.				*Caridina longshan*	16,853	OP177695
24.				*Stygiocaris lancifera*	15,750	PP059120
25.		Bresilioidea	Alvinocarididae	*Alvinocaris chelys*	15,910	NC_018778
26.				*Alvinocaris longirostris*	16,021	OR570908
27.				*Microcaris indica*	15,922	NC_054368
28.				*Nautilocaris saintlaurentae*	15,928	NC_021971
29.				*Rimicaris kairei*	15,901	OR570909
30.				*Shinkaicaris leurokolos*	15,903	NC_037487
31.		Nematocarcinoidea	Rhynchocinetidae	*Rhynchocinetes brucei*	16,158	NC_081006
32.		Oplophoroidea	Oplophoridae	*Acanthephyra sp*.	16,205	MT879756
33.		Palaemonoidea	Palaemonidae	*Macrobrachium nipponense*	15,783	PP747074.
34.				*Palaemon modestus*	15,734	NC_082428
35.				*Anchistus australis*	15,396	NC_046034
36.				*Gnathophyllum americanum*	15,842	NC_086964
37.				*Macrobranchium rosenbergii*	15,766	KY865098
38.				*Macrobrachium hainanense*	15,782	PP747075
39.		Pandaloidea	Pandalidae	*Procletes levicarina*	15,889	NC_081992
40.				*Plesionika edwardsii*	15,956	NC_068072
41.		Penaeoidea	Penaeidae	*Penaeus vannamei*	15,990	NC_009626
42.				*Penaeus indicus*	16,071	KX462904
43.	Brachyura	Portunoidea	Portunidae	*Scylla serrata*	15,775	NC_012565
44.				*Scylla tranquebarica*	15,833	NC_012567
45.	Stenopodidea		Spongicolidae	*Spongicola levigatus*	15,901	KU188325
46.				*Stenopus hispidus*	15,528	NC_018097

*Note:* The highlighted individual has been sequenced in this study.

## 4 Discussion

The *Potamalpheops sp*. is one of the new species found from southern Taiwan. According to the morphological evidences and sequence similarity index of COI gene, the specimen that obtained was identified as *Potamalpheops sp*. (Marin 2021). There were only three sequences of *Potamalpheops sp*. submitted to NCBI from Vietnam (Accession: MW808997, MW803170, MW803169) (Marin 2021) and the sequence is also matched to other shrimps in the family Alpheidae (NC_038116). A very few species has been identified so far based on the morphology but there still needs molecular data to support this widely distributed species genetic stock (Anker 2005). The mitogenome sequencing reveals a 16,605 bp of circular genome for the *Potamalpheops sp*. which belongs to the family Alpheidae. The circular mitogenome is having a similar pattern of 37 genes as found in most of the crustaceans. This species is having a 48 bp intergenic spacer between 16S rRNA and D‐loop also, a variety of intergenic spacer and overlaps seen in the diverse family of Alpheidae. Also, most of the spacers and overlaps can be seen in *Alpheus randalli* and *Alpheus bellulus* , where almost all gene shows either an overlap or spacer (Wang et al. 2020). The same pattern can be seen in *Synalpheus microneptunes* (Chak, Barden, and Baeza 2020). A substantial A+T bias was observed in the mitogenome of *Potamalpheops sp*. (65.1%) it is in accordance with many species of family Alpheidae (Chak, Barden, and Baeza 2020; Ennis et al. 2021; Shen et al. 2012). A substantial A+T bias was observed in the mitogenome of *Potamalpheops sp*. (65.1%) it is in accordance with many species of the family Alpheidae (Chak, Barden, and Baeza 2020; Ennis et al. 2021; Shen et al. 2012). The percentage of A+T composition can significantly differ among various genes. The AT percentage is highly influencing on the stability and replication of the mitogenome and it greatly helps in the environmental adaptation. The AT and GC‐skew values were employed to characterize the compositional bias between the two strands (Perna and Kocher 1995). The current species shows some incomplete and truncated stop codons which is in agreement with many crustacean species. Crustacean mitochondrial genomes frequently exhibit truncated or shortened protein due to occurrence of premature stop codons, which are supposed to be resolved through post‐transcriptional poly‐adenylation (Beckenbach 2009; Chak, Barden, and Baeza 2020; Ivey and Santos 2007; Tan et al. 2019). The codon usage in PCGs is important for regulating gene expression and also influenced by translational selection (Akashi 1994; Siddika et al. 2024).

The degeneracy of the genetic code allows many synonymous codons to transcribe most amino acids (Athey et al. 2017; Siddika et al. 2024). Synonymous codons are spontaneously produced at varying rates by various animals. The choice of codons utilized may have an effect on the expression, structure, and function of proteins (Athey et al. 2017). If there is no bias in codon usage, the RSCU value would be 1.0. When codons are used more frequently than expected, their RSCU values are over 1.0, while when they are used less frequently than expected, their RSCU values are below 1.0. In the present study the RSCU values ranged between 0.32 (Ala) and 1.94 (Ser1). Also, some amino acids were utilized by maximum four codons and least codons used are two and this is similar pattern seen in many crustaceans (Chak, Baeza, and Barden 2020; Shen et al. 2012).

In *Potamalpheops sp*. the tRNA cloverleaf secondary structure, which lacks the usual DHU arm in tRNAS1 is also seen in many crustaceans as well as in metazoans. The tRNA‐Ser1 gene was missing the stem and loop structure of the pseudouridine arm, often known as the T‐arm. tRNA arm deletions, either complete (stem and loop) or partial (loop only), have been observed in different decapod crustaceans (Baeza 2018; Chak, Barden, and Baeza 2020; Ivey and Santos 2007; Li et al. 2016; Tan et al. 2019). The tRNA mentioned, as well as their function, can be further understood by referring to the sources cited. Additionally, the role of these truncated tRNA molecules may be supported by elongation factors which can be complemented by aminoacylation (Watanabe, Suematsu, and Ohtsuki 2014). The position and alignment of the rRNA genes differ from the typical arrangement found in Pancrustaceans (Miller et al. 2004). Comparing insects and crustaceans share the GA‐block, an extremely conserved element whose function is still a mystery (Kuhn, Streit, and Schwenk 2008; Liu and Cui 2010; Zhang and Hewitt 1997). Also, there has been many events of loss of GA blocks or replaced with poly Gs has been reported in insects and Daphnia (Liu and Cui 2010). Along the whole length of this intergenic region, a visual examination of this non‐coding area showed many mono‐nucleotide adenine and thymine repeats. Regarding location and conservation, it is believed that the stem‐loop structure observed in insects and decapods is also present here, potentially serving a similar function. The putative D‐Loop/CR in crustaceans has only been described in a small number of articles, with few citations. This lengthy non‐coding area seems to be somewhat ordered in certain species, such as the decapod *Panulirus stimpsoni* (Chinese spiny lobster) (Liu and Cui 2011) and the non‐decapod branchiopod genus Daphnia (Kuhn, Streit, and Schwenk 2008). The D‐Loop/CR is not clearly organized in *Potamalpheops sp*., although it is in other species (Baeza 2018; Chak, Barden, and Baeza 2020).

Also, the selection pressure analysis has been showed the Ka/Ks (non‐synonymous substitution/synonymous substitution) ratio of 13PCGs along the superfamily alphoidea. This estimates the natural selection patterns where shows an overall purifying selection/negative/ stabilizing selection. The similar trend of purifying selection is found in *S. microneptunus* , *A. japonicus* , *A. distinguendus* , *M. rosenbergii* , *M. nipponense* , *M. lanchesteri* (Chak, Barden, and Baeza 2020; Shen et al. 2012). The study of selective pressure in mitochondrial PCGs in crustaceans has been limited. However, research in other arthropods, such as decapod crustaceans, has shown a consistent pattern of persistent purifying selection in mitochondrial PCGs. Several studies have also been documented this kind of pattern, as referenced in Caribbean spiny lobster, Rhyparochromidae (Baeza 2018; Chak, Baeza, and Barden 2020; Li et al. 2016). Curiously, in all the Alphoeidea, the genes COI and ND2 displayed higher ratios of Ka/Ks compared to other genes, nonetheless the values were still below 1, suggesting that these two genes experience lower levels of selective pressure compared to the other PCGs, this results are also corroborate with the conserve site analysis of nucleotide and genetic distance of genes (Shen et al. 2012). Additionally, it was discovered that these two genes had greater ratios of Ka/Ks compared to other mitochondrial genes in the genus Alpheus (Shen et al. 2012). This implies that the genes in Alpheidae may experience reduced selective pressures that might be an effect of preserve genetic diversity by removing the harmful mutations.

There is collective evidence claiming that crustaceans and hexapods belong to the same clade, known as Pancrustacea. Additionally, they share a common mitochondrial gene order, referred to as the Pancrustacean ground pattern (Shen et al. 2012). The gene order discovered in *Potamalpheops sp*. is similar with some exceptions as that described for the majority of caridean shrimps (Tan et al. 2015) and aligns with the assumed Pancrustacean (Hexapoda + Crustacea) ancestral pattern (Tan et al. 2017). There has been some novel re‐arrangement of genes in *Potamalpheops sp*. seen which makes this species different from the other closely related genus *Alpheus* and *Synalpheus* (Chak, Barden, and Baeza 2020; Qian et al. 2011; Shen et al. 2012; Tan et al. 2019; Wang et al. 2020; Zhong, Zhao, and Zhang 2019).

The transposition of these genes has not been documented in any other crustacean mitochondrial genomes that have been examined thus far. There is a notion suggesting that rearrangements in the mitogenome may happen due to recombination, but recombination in animal mitogenomes is typically infrequent (Tsaousis et al. 2005). Furthermore, the potential rearrangements could be attributed to unidentified compensating mechanism resulting in a substantial reduction of gene products. However, a duplication event for CR has been found in *A. randalli* , deletion of CR in *L. forceps* and transposition as well as reversal has been shown in *S. microneptunus* mitogenome (Chak, Barden, and Baeza 2020; Wang et al. 2020). The mechanism of “duplication/random loss” is widely accepted as the leading hypothesis for explaining gene translocation in mitogenomes. This mechanism involves the duplication of gene regions through slipped‐strand mispairing during replication, followed by the subsequent deletion of one of the duplicated regions (Boore 2000; Shen et al. 2012). Another proposed possibility is the mechanism of “intramitochondrial recombination,” which includes the targeted breaking and rejoining of DNA double strands. This process promotes gene rearrangements and gene inversions (Dowton and Campbell 2001). We propose that the latter mechanism might be the one. The transposition of the tRNA‐Phe and tRNA‐Ser1 along with 16S rRNA and 12S rRNA gene and reversal in tRNA‐Ser1, tRNA‐Pro, tRNA‐Ile in the *Potamalpheops sp*. mitochondrial genomes is controlled by a specific mechanism. Further investigation into the many evolutionary pathways responsible for mitochondrial gene rearrangements in closely related species is warranted. Nevertheless, the question of whether mitochondrial gene synteny may be used to uncover genealogical relationships among Caridea and other Decapod crustaceans has yet to be answered.

The mitochondrial sequence is a commonly employed molecular marker for making inferences about the evolutionary relationships of animals (Zardoya and Meyer 1996). The phylogenetic tree inferred a closed relation between the family Alpheidae. The current species of interest made a highly similar pattern to the all Alpheidae and made its presence in the family. The relationship validates the monophyly of the Alpheidae family, which aligns with findings from prior research that evaluated a combination of mitochondrial and nuclear genes or concentrated on mitochondrial PCGs albeit with a smaller sample size of caridean shrimps. Additionally, established clades within the infraorder Caridea include the Alvinocaridae, Atyidae, Palaemonidae, and Pandalidae families. The Maximum Likelihood analyses provided support for monophyly of these caridean families. However, the relationships among the families were found to be influenced by the optimality criteria used. A recent phylogenomic study provided additional support for the observed sister relationship between the families Palaemonidae and Alpheidae (Chak, Barden, and Baeza 2020; Ennis et al. 2021; Shen et al. 2012; Wang et al. 2020; Wolfe et al. 2019). The phylogenetic tree from the analyses were corroborated by robust statistical values. Typically, all the family belongs to infraorder Caridea are grouped together along with the crab species belong to the infraorder Brachyura. Also, the superfamily Penaeoidea with the family Penaeidae shows a peculiar grouping with the outgroup. Our research indicates that mitochondrial genomes provide enough phylogenetic data to determine the monophyly of higher taxa within the Caridea. This is specifically at the superfamily and family levels. Nevertheless, it is crucial to acknowledge that the relationships between these monophyletic clades may be influenced by the selection of genetic markers and the specific techniques employed for reconstruction.

Lastly, the first ever complete mitogenome was identified for the purple zebra shrimp, *Potamalpheops sp*. The genome is 16,605 base pairs in length and has a circular structure. It shares the same gene composition as other multicellular animals. Nevertheless, the gene order deviates from the presumed typical gene structure of Caridea and all other alpheid mitogenome that have been sequenced so far. It seems that four genes have been moved to different locations, and three other genes have also seen a change in their orientation. Both the processes of “duplication/random loss” and “intramitochondrial recombination” could potentially be accountable for these rearrangements. The present individual also showing a purifying selection toward caridea infraorder. The study determined the taxonomic classification of this species within the family by examining protein coding genes that undergo purifying selection. Additionally, a thorough genomic analysis needs to explore the use of additional mitochondrial areas for species identification and population genetics studies. Since the study only focuses on the mitochondrial genome, further research on nuclear genome‐wide studies has the ability to definitively address additional uncertainties regarding this shrimp family.

## Author Contributions

**Sofia Priyadarsani Das:** conceptualization (lead), data curation (lead), formal analysis (lead), investigation (supporting), methodology (lead), resources (equal), software (lead), validation (lead), visualization (lead), writing – original draft (lead), writing – review and editing (lead). **Yu‐Kai Kao:** formal analysis (supporting), resources (equal), writing – original draft (supporting), writing – review and editing (supporting). **Huu‐The Nguyen:** writing – original draft (supporting), writing – review and editing (supporting). **Yu‐Ru Lin:** formal analysis (supporting), writing – original draft (supporting), writing – review and editing (supporting). **Zhen‐Hao Liao:** formal analysis (supporting), writing – original draft (supporting), writing – review and editing (supporting). **Yeh‐Fang Hu:** investigation (lead), project administration (supporting), supervision (supporting), writing – original draft (supporting), writing – review and editing (supporting). **Fan‐Hua Nan:** funding acquisition (lead), project administration (lead), supervision (lead), writing – original draft (supporting), writing – review and editing (supporting).

## Conflicts of Interest

The authors declare no conflicts of interest.

## Data Availability

Complete mitochondrial genomes assembled and annotated in the present study are available on the NCBI GenBank Database with the Accession Number OR825035.
